# Severe Hypothyroidism Complicated by Myopathy and Neuropathy with Atypical Demyelinating Features

**DOI:** 10.1155/2021/5525156

**Published:** 2021-05-19

**Authors:** Malgorzata Monika Brzozowska, Shraddha Banthia, Simon Thompson, Manisha Narasimhan, James Lee

**Affiliations:** ^1^Department of Endocrinology, Sutherland Hospital, Sydney, NSW, Australia; ^2^The University of New South Wales Sydney, Faculty of Medicine, Sydney, NSW, Australia; ^3^Department of Chemical Pathology, NSW Health Pathology, Prince of Wales Hospital, Sydney, NSW, Australia; ^4^Department of Neurology, Sutherland Hospital, Sydney, NSW, Australia; ^5^The University of Sydney, Brain and Mind Centre, Sydney, NSW, Australia; ^6^Department of Neurology, Royal North Shore Hospital, Sydney, NSW, Australia

## Abstract

Autoimmune hypothyroidism may result in a wide range of neuromuscular disorders. The frequently observed neurological manifestations of acquired hypothyroidism include mild to moderate myopathy and sensorimotor neuropathy, which usually resolve by clinical and electrophysiological criteria, in adults treated with thyroid hormone replacement. We report a case of a 30-year-old male with severe hypothyroidism secondary to chronic autoimmune thyroiditis who presented with a 2-year history of progressive fatigue, upper and lower limb weakness, myalgia, and intermittent paraesthesia. His neurological exam demonstrated proximal and distal muscle weakness, lower limb areflexia, and relatively intact sensory modalities. The patient's biochemistry revealed unusually and profoundly raised the thyroid stimulating hormone (TSH) level of 405.5 mIU/L (reference range (RR): 0.27–4.2 mIU/L) and creatine kinase (CK) level of 20,804 U/L (RR: 45–250 U/L), while his nerve conduction studies (NCS) demonstrated severe sensorimotor polyneuropathy with both axonal and demyelinating features. Thyroid hormone replacement therapy over the first 3 months resulted in biochemical normalization of his extremely deranged thyroid function tests (TFTs) and CK levels. At 12 months, despite maintaining euthyroidism and noticeable improvement in strength, his nerve conduction studies (NCS) demonstrated the continued absence of distal motor and sensory responses in his lower limbs with only partial improvement in sensory amplitudes and conduction velocities in his upper limbs. This report highlights the potential for severe neuromuscular consequences from advanced and chronic autoimmune hypothyroidism. The patient's myopathy has resolved over a period of three months with prompt normalization of CK levels. Concerningly, the patient achieved significant but incomplete recovery from his mixed axonal and demyelinating neuropathy with residual mild distal weakness and areflexia in his lower limbs and persistent motor and sensory impairments on his NCS. The severity and incomplete resolution of our patient's neurological manifestations emphasize the importance of early diagnosis and the need for prompt therapeutic intervention for hypothyroidism.

## 1. Introduction

Chronic autoimmune thyroiditis (also known as Hashimoto's disease) is the most common cause of hypothyroidism in iodine-sufficient areas [1]. Neurological features of hypothyroidism include myopathy, mono- and polyneuropathy, cognitive impairment, rarely cerebellar ataxia, and in more extreme presentations, myxoedema coma. The prevalence of neuromuscular manifestations associated with thyroid disorders has varied significantly between studies [2]. Myopathy is a frequent manifestation of hypothyroidism (between 20% and 80% of reported cases), manifesting clinically as weakness, cramps, and myalgias often associated with mild to moderate elevations in creatine kinase [2, 3]. The estimates of neuropathy in hypothyroidism range between 10% and 70% [2] with nerve conduction studies in hypothyroid neuropathy most commonly demonstrating a mild sensory axonal neuropathy [2, 4]. We present a patient with severe hypothyroidism due to Hashimoto's disease, with associated rhabdomyolysis complicated by severe mixed axonal and demyelinating polyneuropathy, with significant but incomplete response to levothyroxine treatment at 12 months after initial presentation.

## 2. Case Presentation

A 30-year-old male presented with a 2-year history of insidious and progressive fatigue, generalised weakness, myalgias, muscle cramps, and intermittent paraesthesiae in his upper and lower limbs. The patient reported impaired concentration and low mood due to mild depression, and he denied any alcohol intake or recreational drug use. The patient's father and his brother were subsequently diagnosed with Hashimoto's thyroiditis.

### 2.1. Examination and Investigations

The patient had loss of hair at the outer third of the eyebrows as well as over his shins, minimal periorbital oedema, and yellow palmar discolouration consistent with hypercarotenemia and macroglossia, with an absence of a palpable goitre or thyroid nodules. The patient's voice was hoarse in quality with slow speech and a delayed response latency when asked questions. There was no evidence of hepatosplenomegaly, lymphadenopathy, or skin hyperpigmentation. Neurological exam demonstrated proximal muscle weakness of grade 4/5 with an inability to rise from a seated position, as well as distal weakness of grade 3/5 in his lower limbs with significant weakness of ankle dorsiflexion bilaterally and absent ankle and knee jerk reflexes. In contrast, surprisingly, all sensory modalities (pinprick, vibration, light touch, and proprioception) appeared relatively spared by clinical examination. He did, however, have a wide-based high stepping gait with bilateral foot drop.

Initial laboratory results (September 2019) showed significantly deranged thyroid function tests (TFT) with an elevated thyroid stimulating hormone (TSH) level of 405.5 mIU/L (reference range (RR): 0.27–4.2 mIU/L), low free thyroxine (fT4) level of 1.6 pmol/L (RR: 12.0–22.0 pmol/L), and undetectable free triiodothyronine (FT3) level < 1.5 pmol/L (RR: 3.1–6.8 pmol/L) with raised thyroid peroxidase (TPO) antibody level of >600 IU/mL, consistent with Hashimoto's thyroiditis. The TSH level was measured by immunoassay with electrochemiluminescent detection (Roche Diagnostics, North Ryde, NSW, Australia). The assay is a sandwich immunoassay with a linear range between 0.005 mIU/L and 100 mIU/L. As TSH level of 405.5 mIU/L was significantly beyond the assay's analytical range, the examined sample required additional dilution in a validated matrix to obtain a numerical result. The initial creatine kinase (CK) level was elevated at 20,804 U/L (RR: 45–250 U/L); however, he had normal renal function. The patient's serial measurements of folate levels have remained within midnormal range with values up to 45.5 nmol/L (05/06/20). Further investigations (September 2019) revealed negative autoimmune screening including ANA, dsDNA, ENA, antinuclear antibodies, myositis-specific antibodies, myositis-associated antibodies, antimyelin-associated glycoprotein, and acetylcholine receptor antibodies. He had normal levels. There was a possible mild vitamin B12 deficiency with a holotranscobalamin level of 42.8 pmol/L (RR: 50.1–165 pmol/L). There was no evidence of monoclonal paraprotein on serum protein electrophoresis (EPG) (03/10/19). The patient's initial biochemistry is given in Table 1 with boldface highlighting abnormal results.

The patient's thyroid ultrasound (September 2019) revealed a normal-sized thyroid gland with a 9 mm nodule in the right lobe confirmed on fine needle aspiration biopsy to be lymphocytic thyroiditis. His nerve conduction studies (NCS) and electromyography (EMG) (18 September 2019) demonstrated an absence of distal lower limb motor and sensory responses and markedly prolonged upper limb distal motor latencies and conduction velocities, sensory nerve conduction velocities, and F-wave latencies. EMG confirmed acute denervation changes in distal muscles alongside widespread chronic partial denervation and reinnervation. The overall pattern was of severe generalised length-dependent sensorimotor neuropathy with both axonal and demyelinating features. An MRI of the brain (November 2019) was normal with no evidence of demyelinating, atrophic, or ischaemic features.

### 2.2. Treatment and Outcomes

**Figure 1:**
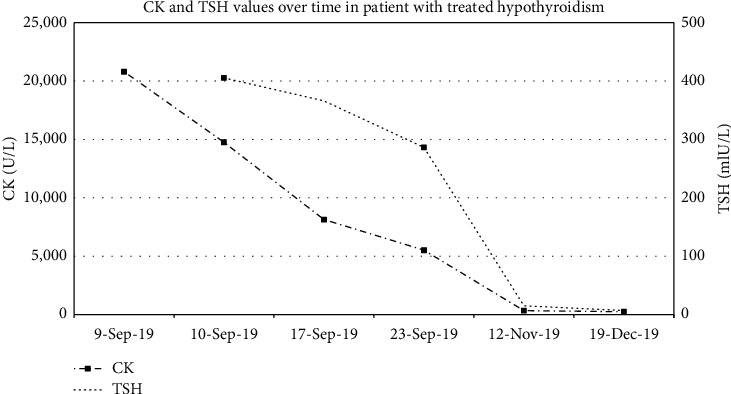
CK and TSH values over time in patient with treated hypothyroidism.

The patient's clinical findings were suggestive of hypothyroidism-associated myopathy and polyneuropathy. He was commenced on levothyroxine at an initial dose of 100 *µ*g daily, with partial and gradual improvement in his neurological presentation. He was also administered vitamin B12 replacement. Following 2.5 weeks of thyroxine replacement, the patient's TSH level had decreased to 183 mIU/L with a decline in CK level to 3203 U/L (Figure 1), and improvement in his proximal strength was observed to grade 5/5 power. There was persistent distal lower limb weakness with bilateral footdrop. His ankle and knee reflexes remained absent.

At two months post levothyroxine initiation (November 2019), his TFT had significantly improved with the TSH level of 15.1 mIU/L, fT4 of 18.7 pmol/L, and fT3 of 5.1 pmol/L with normalization of his liver function tests, likely due to resolved rhabdomyolysis and improved total vitamin B12 levels to 266 pmol/L (RR: 150–700). Repeat NCS at this time continued to demonstrate severe sensorimotor neuropathy with mixed axonal and demyelinating features. After three months of thyroxine replacement, the patient reported improvement in his lower limb muscle strength and gait, mood and concentration, energy levels, and in his speech quality. There was persistent distal lower limb weakness and lower limb areflexia.

**Figure 2:**
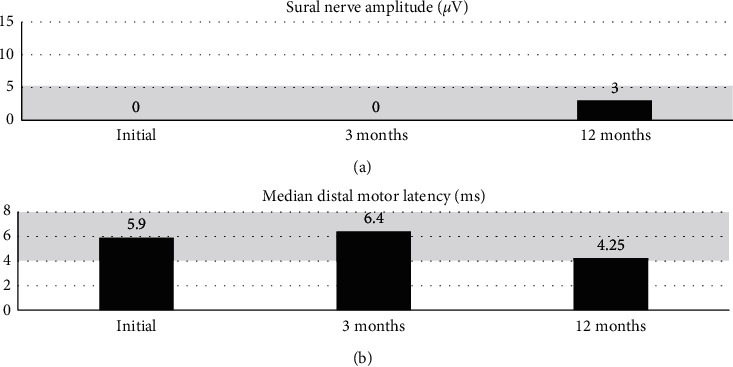
Serial nerve conduction values over time. (a) Sural nerve amplitude (*μ*V). (b) Median distal motor latency (ms).

Follow-up clinical assessment at 12 months after his diagnosis (September 2020) revealed significant improvement in lower limb strength with persistent areflexia. Congruently, with physical findings, his repeat NCS revealed some improvement in sensory amplitudes and conduction velocities in patient's arms; however, motor and sensory responses in his legs remained largely absent (Figure 2).  Figure 2(a) demonstrates the trend of sural sensory nerve amplitudes, whilst Figure 2(b) depicts median distal motor nerve latency over three studies performed at baseline, 3 months, and 12 months after diagnosis. The abnormal ranges for these values are shaded in grey, with values trending towards normal at the 12-month study.

## 3. Discussion

The neuromuscular manifestations of hypothyroidism frequently occur in conjunction with common systemic features such as fatigue, weight gain, cold intolerance, and dry skin [1]. Rarely, as in the presented case, muscle and nerve dysfunction may flag the presence of hypothyroidism, highlighting the importance of considering thyroid dysfunction in any patient presenting with new neuromuscular symptomatology [2].

The pathophysiology of hypothyroid myopathy, although incompletely understood, is thought to be secondary to an altered metabolic state, which results in aberrant glycogen and oxidative metabolism in the actin-myosin unit [3, 5]. Severe or prolonged oxidative damage causes muscle cell injury and rhabdomyolysis. The muscle involvement in hypothyroidism ranges from asymptomatic CK elevation to proximal myopathy mimicking inflammatory myositis and rarely, frank rhabdomyolysis [6, 7] and necrotising myopathy [8]. Furthermore, patients with primary hypothyroidism are more at risk of developing statin-induced myopathy [9].

Mononeuropathy affecting the median nerve (i.e., carpal tunnel syndrome) is the most common peripheral neurological complication of hypothyroidism [4]. Hypothyroid-associated polyneuropathy presents commonly with length-dependent symmetric sensory neuropathy and predominantly axonal neuropathy [2, 4] without unique distinguishing features [10]. Interestingly, although polyneuropathy occurs commonly in hypothyroidism, at times, it may not be detected on clinical exam unless diagnosed via electrophysiological criteria [11].

The physiological mechanisms involved in polyneuropathy due to hypothyroidism are not well understood but are possibly related to axonal degeneration, deposition of mucopolysaccharides in the endoneurial interstitium and perineurial sheath with segmental demyelination and remyelination changes [11], and metabolic alterations affecting Schwann cells. Electrophysiological studies have reported resistance to the conduction of impulses with increased response latencies and decreased conduction velocity.

Our patient presented with severe neuropathy with mixed axonal and demyelinating features including electrodiagnostic findings suggestive of demyelination on his NCS. While metabolic neuropathies are usually thought to be axonal, demyelinating neuropathies are less frequently documented in the literature in association with hypothyroidism [12, 13].

Our patient's repeat NCS results, which were expected to improve following thyroid hormone replacement [10], remained unchanged at 2 months after the diagnosis of hypothyroidism. Despite achieving and maintaining clinical euthyroidism and significant improvement in muscle strength, his NCS markers only partially corrected after 12 months of thyroid hormone replacement. Furthermore, vitamin B12 levels at the time of repeat NCS testing were replete, making vitamin B12 deficiency an unlikely contributor to his presentation.

The literature highlights an association between the duration of hypothyroidism and hypothyroid-related neuropathy [14]. Furthermore, previous studies have shown that peripheral neuropathy due to hypothyroidism, diagnosed by electrophysiological criteria, occurs early in the course of thyroid disease and initially remains latent and asymptomatic [11, 15, 16]. The presented patient had significant neuropathy with persistent distal weakness and bilateral footdrop gait. His profound elevations of TSH levels, to values which are rarely seen in clinical practice, are suggestive of a prolonged period of overt hypothyroidism, possibly accounting for the severity of our patient's electrodiagnostic findings and his persistent neurological deficit. Importantly, in previous studies, over one-third of patients affected by hypothyroidism had residual polyneuropathy after 12 months of adequate thyroid replacement therapy [2], with a reported case of severe hypothyroid neuropathy which had only resolved after 6 years of thyroid hormone replacement [17].

Previous reports have pointed to an association between Hashimoto's thyroiditis and autoimmune demyelinating neuropathies such as Guillain–Barré syndrome, chronic inflammatory demyelinating polyneuropathy, and multifocal motor neuropathy, likely due to an underlying shared genetic susceptibility to autoimmune conditions [18–22]. The significant clinical improvement in our patient's distal weakness together with partial improvement in his NCS findings after 12 months of thyroxine therapy and the absence of other systemic involvements make the presence of an autoimmune demyelinating neuropathy a less likely diagnosis. Furthermore, an absence of hepatosplenomegaly, lymphadenopathy, skin hyperpigmentation, clonal plasma cell disorder, or lymphoproliferative disease excluded the presence of POEMS syndrome [23].

The majority (57–90%) of individuals with hypothyroidism present with mild to moderate elevations of serum creatine kinase (CK) to values less than 5000 U/L [24]. The reported patient presented with CK values more than one hundred times the reference range (RR: 45–250 U/L), likely consistent with rhabdomyolysis [25] and more commonly described in immune-mediated myopathies. The detailed laboratory evaluation of inflammatory myopathies was negative, excluding other myopathic disorders. Previous case studies of patients with hypothyroid-induced myopathy have shown that successful treatment with thyroxine therapy results in clinical and biochemical recovery [24, 26]. In the present case, following the resolution of hypothyroidism, the patient's CK level had normalised with a complete restoration of power in his proximal muscles.

An interesting aspect of this case is the biochemical severity of patient's hypothyroidism. Importantly, we have obtained a numerical result of patient's TSH concentration which allowed for accurate biochemical assessment of the patient's response to therapy.

When faced with extreme and unusual laboratory results, it is important to consider the possibility of analytical interferences. Interferences with immunoassays are common and are easily missed unless clinical and laboratory personnels are vigilant when reviewing biochemistry results. Common causes of TSH immunoassay interferences include the presence of cross-reacting (heterophile) antibodies, exogenous biotin in the sample, and macroanalyte complexes (analyte molecules bound together by immunoglobulin) which have delayed clearance from the plasma and therefore are present in higher concentrations [27]. In the present case, the pattern of exceedingly high TSH values with low concentrations of both thyroid hormones, positive anti-TPO antibody status, and clinical features consistent with a severe hypothyroid state improved the confidence in the analytical accuracy of the results.

In conclusion, the presented case highlights the severe neuromuscular consequences of profound and likely longstanding chronic autoimmune hypothyroidism. The patient presented with severe myopathy, which resolved over a period of two months with the introduction of thyroxine therapy. Despite early diagnosis and successful treatment of the biochemical hypothyroidism, the patient achieved only partial recovery from his sensorimotor peripheral neuropathy with residual distal weakness and areflexia in his lower limbs and ongoing motor and sensory impairments on his NCS at 12 months. The severity and incomplete resolution of the patient's neurological manifestations emphasises the importance of early diagnosis and prompt therapeutic intervention for hypothyroidism.

The routine screening of patients presenting with neuromuscular symptoms for thyroid dysfunction would lead to timely administration of thyroid replacement therapy with a subsequent reduction in their serious neuromuscular comorbidities. Importantly, physicians need to be aware about long-term neuromuscular sequelae which may complicate untreated hypothyroidism. Therefore, we hope to increase diagnostic awareness for an early recognition and treatment of neurological complications arising from thyroid hormone deficiency.

## Figures and Tables

**Table 1:** Results of initial laboratory tests (September 2019).

Parameters	Parameter value	Reference interval
Sodium (mmol/L)	140	135–145
Potassium (mmol/L)	4.0	3.5–5.2
eGFR (mL/min/1.73 m^2^)	>90	>90
Fasting glucose (mmol/L)	3.9	3.0–5.5
Albumin (g/L)	46	33–48
Alanine aminotransferase (U/L)	**197**	<51
Aspartate aminotransferase (U/L)	**345**	<36
Alkaline phosphatase (U/L)	45	30–110
*γ*-Glutamyl transferase (U/L)	12	5–50
Bilirubin (*μ*mol/L)	6	0–20
Corrected calcium (mmol/L)	2.3	2.1–2.6
Magnesium (mmol/L)	0.95	0.7–1.1
Phosphate (mmol/L)	1.25	0.75–1.5
Holotranscobalamin (pmol/L)	**42.8**	50.1–165
Folate (nmol/L)	19.3	8.83–60.8
Creatine kinase (U/L)	**20,804**	45–250
Total cholesterol (mmol/L)	**7.9**	3–5.5
Free T4 (pmol/L)	**1.6**	12–22
Free T3 (pmol/L)	<1.5	3.1–6.8
TSH (mIU/L)	**405.5**	0.27–4.2
Haemoglobin (g/L)	142	130–180
Platelets (×10^9^/L)	270	150–450
White cell count (×10^9^/L)	7.67	3.5–11
Thyroid peroxidase (TPO) antibodies (IU/mL)	**>600**	≤35
Thyroglobulin antibodies (IU/mL)	**152**	0–115
Intrinsic factor antibodies	Negative	
Gastric parietal cell antibodies	Negative	
Antinuclear antibodies	Negative	
Double stranded DNA antibodies	Negative	
ENA screen	Not detected	
Myositis antibodies screen	Not detected	
Antimyelin-associated glycoprotein	Not detected	
Acetylcholine receptor antibodies	Not detected	

Values in bold represent abnormal results
